# A Pan-Cancer Analysis of the Oncogenic Role of Nuclear Transport Factor 2 in Human Cancers

**DOI:** 10.3389/fonc.2022.829389

**Published:** 2022-01-28

**Authors:** Yu Li, Yongsheng Huang, Shuwei Ren, Xing Xiao, Haotian Cao, Juan He

**Affiliations:** ^1^ Department of Laboratory Medicine, Peking University Shenzhen Hospital, Shenzhen, China; ^2^ Cellular & Molecular Diagnostics Center, Sun Yat-Sen Memorial Hospital, Sun Yat-Sen University, Guangzhou, China; ^3^ Guangdong Provincial Key Laboratory of Malignant Tumor Epigenetics and Gene Regulation, Guangdong-Hong Kong Joint Laboratory for RNA Medicine, Medical Research Center, Sun Yat-Sen Memorial Hospital, Sun Yat-Sen University, Guangzhou, China; ^4^ Guangdong Provincial Key Laboratory of Colorectal and Pelvic Floor Diseases, Sun Yat-Sen University Sixth Affiliated Hospital, Sun Yat-Sen University, Guangzhou, China; ^5^ Department of Dermatology, Shenzhen Children’s Hospital, Shenzhen, China; ^6^ Department of Oral and Maxillofacial Surgery, Sun Yat-Sen Memorial Hospital, Sun Yat-Sen University, Guangzhou, China; ^7^ Department of Rheumatology and Immunology, Peking University Shenzhen Hospital, Shenzhen, China; ^8^ Shenzhen Key Laboratory of Immunity and Inflammatory Diseases, Shenzhen, China

**Keywords:** NUTF2, pan-cancer, prognostic, tumor-infiltrating lymphocyte, CAFs

## Abstract

Nuclear transport factor 2 (NUTF2) is a GDP-binding protein that participates in the nucleocytoplasmic transport process. The role of NUTF2 in cancer development is largely unknown and lacks systemic assessment across human cancers. In this study, we performed a pan-cancer analysis of NUTF2 in human cancers. Out of 33 types of cancers, 19 types had significantly different expression of NUTF2 between tumor and normal tissues. Meanwhile, survival analysis showed that NUTF2 could be an independent prognostic factor in several tumor types. Further analysis suggested that the expression of NUTF2 expression was correlated with the infiltration of immune cells, such as CD8^+^ T cells, effector memory CD4^+^ T cells, and cancer-associated fibroblasts in kidney renal clear cell carcinoma. Moreover, co-expression analysis showed the positive association between NUTF2 and cell proliferation biomarkers (MKI67and PCNA) and epithelial–mesenchymal transition markers (VIM, TWIST1, SNAI1, SNAI2, FN1, and CDH2), suggesting that NUTF2 plays important roles in regulating cancer proliferation and metastasis. This pan-cancer analysis of NUTF2 provides a systemic understanding of its oncogenic role across different types of cancers.

## Introduction

Despite the improving capacity of diagnosis and therapy, cancer remains the second leading cause of death worldwide. The GLOBOCAN predicts that there will be 27.5 million new cancer patients worldwide in 2040, with an increase of 61.7% from 2018 (when the number of new cancer cases was 18.1 million) (1). Genomic and epigenomic studies have significantly demonstrated that biological heterogeneity is a central property of cancers and patients. Furthermore, the same genetic variant may play a different role across various types of cancers (2). Thus, a pan-cancer analysis of cancer-associated genes will be helpful for understanding their roles in cancer development.

Nuclear transport factor 2 (NUTF2, also known as NTF2) is a small GDP Ran-binding protein. It was firstly identified as a nucleocytoplasmic transport enhancer through interaction with nucleoporin FxFG (3, 4). Additionally, NUTF2 was shown to be a GDP-dissociation inhibitor and regulated the GDP-Ran gradient (5–7). Interestingly, a recent study uncovered the capacity of NUTF2 to reduce the nuclear size and diameter of the nuclear pore complex (NPC) (8). It was demonstrated that NUTF2 plays vital roles in the phenotype of eyes and diabetic retinopathy *via* regulating the nuclear import of Ran proteins and the VEGF signaling pathway (9, 10). With respect to cancer, upregulation of NUTF2 was found in glioma tissues and overexpression of NUTF2 promoted migration and proliferation of glioma cells, indicating its oncogenic role in glioma (11). However, the role of NUTF2 in other cancer types is largely unknown.

In the present study, we conducted a pan-cancer analysis of NUTF2 based on the TCGA dataset. NUTF2 expression profile and prognostic significance were investigated among various human cancers. Additionally, genetic alteration, DNA methylation, immune infiltration, and protein interactions were also investigated. Our study comprehensively analyzed the oncogenic role of NUTF2 across various cancer types, and highlights the possibility of NUTF2 to serve as a cancer prognostic biomarker.

## Materials and Methods

### Gene Expression and Survival Analysis

TIMER2.0 online tool (http://timer.comp-genomics.org/) was used to compare the expression of NUTF2 between tumor and adjacent normal tissues across 33 types of cancers (12). We utilized the “Survival Map” module of GEPIA2.0 (http://gepia2.cancer-pku.cn/#index) database to investigate the association between NUTF2 expression and survival status (13). The NUTF2 median expression was set as the cutoff value in determining the high or low expression of NUTF2.

### Genetic Alteration and DNA Methylation Analysis

Genetic alteration analysis of NUTF2 was conducted through the “TCGA Pan-Cancer Atlas Studies” dataset in the cBio Cancer Genomics Portal (http://cbioportal.org) (14). The genetic alteration frequency can be visualized in the “Cancer Types Summary” sub-menu. In order to evaluate the NUTF2 DNA methylation pattern, we used the GSCA (Gene Set Cancer Analysis) (http://bioinfo.life.hust.edu.cn/GSCA/#/) (15) approach to evaluate the impact of the DNA copy number amplification and methylation status on NUTF2 expression.

### Co-Expression Analysis of NUTF2 in Pan Cancers

The “Gene_Corr” module in the TIMER2.0 online resource was applied to investigate the association between NUTF2 expression and proliferation markers (PCNA and MKI67), EMT markers (VIM, TWIST1, SNAI1, SNAI2, FN1, and CDH2), and immune marker gene sets (CD86, CSF1R, CCL2, CD68, IL10, NOS2, IRF5, PTGS2, CD163, VSIG4, and MS4A4a) in various cancer types. Spearman’s correlation test was conducted to calculate the *p*-value. *p* < 0.05 was considered significant.

The “Similar Genes Detection” module in the GEPIA2.0 platform was used to identify the top 200 genes that are most associated with NUTF2 expression. The correlations between NUTF2 and the top 5 genes (COX4NB, E2F4, NAE1, NIP7, and ORC6L) in pan-cancer were calculated in the “Correlation Analysis” module of the GEPIA2.0 online resource.

### Immune Cell Infiltration Analysis

We used the R package “estimate” (16) to calculate the stromal/immune/estimate score of each sample. The correlations between NUTF2 expression and stromal/immune/estimate scores were calculated by Spearman’s test. The association between TILs (tumor-infiltrating lymphocytes) abundance and NUTF2 expression was inferred by using the TISIDB online platform (17). To investigate the impact of NUTF2 on cancer-associated fibroblast infiltration, the TIDE, XCELL, MCPCOUNTER, and EPIC algorithms were performed for immune infiltration estimations. Purity-adjusted Spearman’s rank correlation test was conducted to calculate the *p*-value. *p* < 0.05 was considered significant.

### Protein–Protein Interaction Analysis

The NUTF2 potential binding partners were identified by using the STRING database (18) with the following parameters: meaning of network edges (evidence), active interaction sources (experiments), minimum required interaction score (low confidence), and max number of interactors to show (no more than 50 interactors). By this way, a total of 50 NUTF2 interactors were obtained.

### Functional Analysis of the Co-Expression Genes of NUTF2

A total of 200 genes that most significantly associated with NUTF2 expression were performed by the pathway enrichment analysis *via* DAVID bioinformatic resources (https://david.ncifcrf.gov/tools.jsp) (19, 20). In this way, the underlying biological themes of the top 200 genes can be obtained.

## Results

### Expression Pattern and Survival Analysis of NUTF2

**Figure 1:**
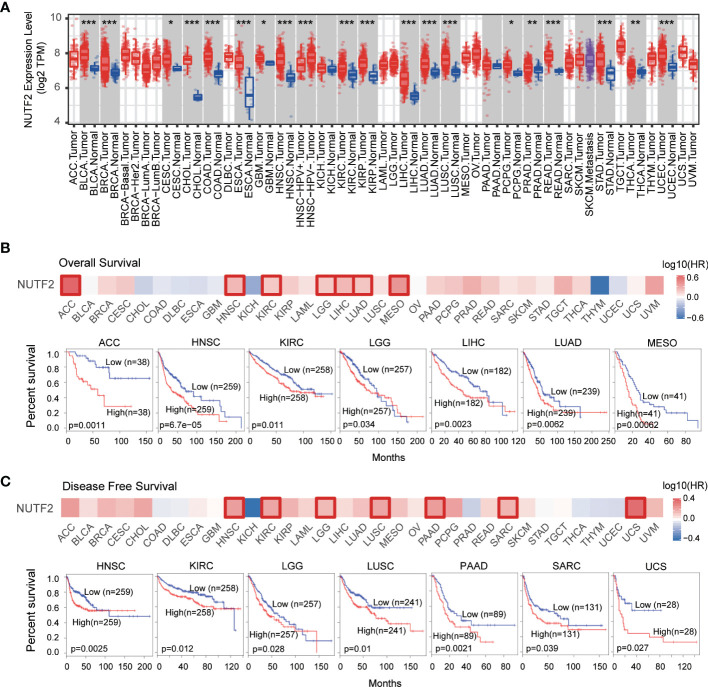
Expression profile and prognostic value of NUTF2 in TCGA cohorts. **(A)** The expression of NUTF2 in different types of cancers or cancer subtypes was analyzed *via* the TIMER2.0 online resource. *p < 0.05; **p < 0.01; ***p < 0.001. **(B)** Overall survival analysis of NUTF2 across the 33 types of cancers. **(C)** Disease-free survival of NUTF2 in different cancer types.

To explore the mRNA expression profile of NUTF2 across all TCGA tumors, we utilized the “Gene_DE” module of the TIMER2.0 web tool. Compared with corresponding adjacent normal tissues, it was found that the expression level of NUTF2 was upregulated in CESC (cervical squamous cell carcinoma and endocervical adenocarcinoma), GBM (glioblastoma multiforme), PCPG (pheochromocytoma and paraganglioma) (*p* < 0.05), PRAD (prostate adenocarcinoma), THCA (thyroid carcinoma) (*p* < 0.01), BLCA (bladder urothelial carcinoma), BRCA (breast invasive carcinoma), CHOL (cholangiocarcinoma), COAD (colon adenocarcinoma), ESCA (esophageal carcinoma), HNSC (head and neck squamous cell carcinoma), KIRC (kidney renal clear cell carcinoma), KIRP (kidney renal papillary cell carcinoma), LIHC (liver hepatocellular carcinoma), LUAD (lung adenocarcinoma), LUSC (lung squamous cell carcinoma), READ (rectum adenocarcinoma), STAD (stomach adenocarcinoma), and UCEC (uterine corpus endometrial carcinoma) tissues (*p* < 0.001) (**Figure 1A**), suggesting the oncogenic role of NUTF2 in these cancers.

We further investigated the prognostic significance of NUTF2 among the 33 different types of cancers. As shown in **Figure 1B**, high NUTF2 expression was correlated with poor overall survival (OS) in ACC, HNSC, KIRC, LGG, LIHC, LUAD, and MESO (*p* < 0.05) (**Figure 1B**). Moreover, the disease-free survival (DFS) analysis suggested that upregulation of NUTF2 was significantly linked to poor prognosis of HNSC, KIRC, LGG, LUSC, PAAD, SARC, and UCS (*p* < 0.05) (**Figure 1C**). These results indicated that NUTF2 is an independent prognostic marker of both DFS and OS in HNSC, KIRC, and LGG.

### Genetic and Epigenetic Alteration Analysis

**Figure 2:**
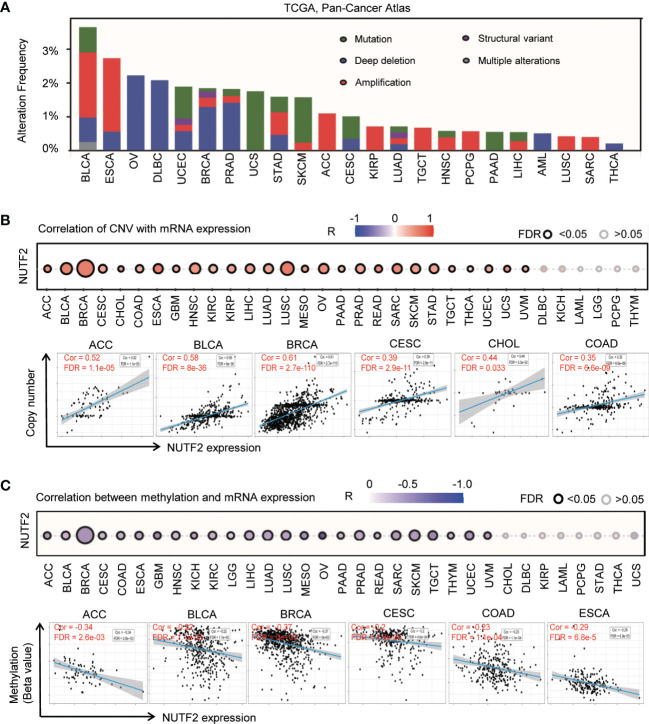
Genetic and epigenetic alteration of NUTF2 in different types of tumors. **(A)** Mutation features of NUTF2 in TCGA tumors using the cBioPortal tool. Structural variation indicates insertion, inversion, translocation, or complex rearrangement of relatively large segments; multiple alterations indicate that two or more genetic alteration types occurred in specific samples. **(B)** The association between copy number variation and NUTF2 expression was analyzed *via* the GSCA approach. **(C)** Correlation between NUTF2 expression level and DNA methylation across 33 types of cancers.

We explored the genetic alterations of NUTF2 in TCGA pan-cancer atlas studies *via* the cBioPortal online resource. It was found that the overall genetic alteration frequency of NUTF2 was relatively low in cancers (**Figure 2A**). The highest alteration frequency of NUTF2 presented in BLCA was 3.65%, and the “copy number amplification” type was the primary form (1.95%). By contrast, no genetic changes were observed in GBM, CRC, UVM, CHOL, KICH, KIRC, MESO, THYM, and LGG. By taking the overexpression of NUTF2 in various types of cancers into consideration, we focused on the copy number amplification variation of the NUTF2 DNA fragment. It was found that higher amplification frequency occurred in ESCA (2.19%), BLCA (1.95%), ACC (1.1%), and KIRP (0.71%). Additionally, we explored the potential associations between copy number amplification and NUTF2 expression across the 33 types of cancers. As shown in **Figure 2B**, we observed a positive correlation among 27 types of cancers (**Figure 2B**) (FDR < 0.05).

Promoter DNA methylation is one of the crucial epigenetic mechanisms for gene expression regulation and cancer progression (21, 22). We used the GSCA (gene set cancer analysis) approach to evaluate the NUTF2 DNA methylation pattern. The significant negative correlation between NUTF2 expression level and DNA methylation was identified in 25 types of cancers (**Figure 2C**) (FDR < 0.05). According to the above data, we reason that DNA copy number amplification and methylation are the two underlying causes of NUTF2 upregulation in cancers.

### Co-Expression Analysis of NUTF2

**Figure 3:**
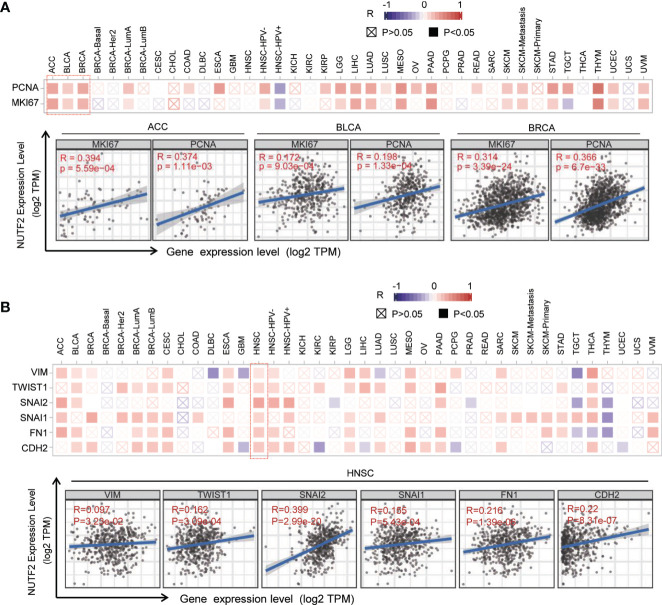
Co-expression analysis between NUTF2 and biomarkers of cell proliferation and EMT markers. **(A)** Associations between NUTF2 expression level and proliferation markers (MKI67 and PCNA) were investigated in different cancer types. **(B)** Correlation analysis on the association between NUTF2 expression and EMT markers (Vimentin, TWIST1, Snail1, Snail2, Fibronectin 1, and CDH2).

To address the possible role of NUTF2 in cancers, gene co-expression network analysis was performed. Gene co-expression analysis is an effective way to delineate gene function and regulatory association (23, 24). In this study, we firstly focused on the potential associations between NUTF2 and the classic proliferation markers, including MKI67 and PCNA (25). As is shown in **Figure 3A**, the corresponding heat map showed that NUTF2 was positively correlated with the expression of MKI67 and PCNA in 16 tumor types, such as ACC, BLCA, and BRCA (*p* < 0.05) (**Figure 3A**). In addition, we also analyzed the correlation between NUTF2 and the epithelial–mesenchymal transition (EMT) markers, Vimentin (VIM), TWIST1, Snail1 (SNAI1), Snail2 (SNAI2), Fibronectin 1 (FN1), and N‐cadherin (CDH2), which were widely accepted to be involved in cancer metastasis (26, 27). Co-expression analysis results indicated that NUTF2 was positively correlated with the expression of these EMT markers among most types of cancers, especially in HNSC (**Figure 3B**). These results suggested that NUTF2 may play an important role in regulating cancer proliferation and metastasis.

### Stromal and Immune Infiltration Analysis

**Figure 4:**
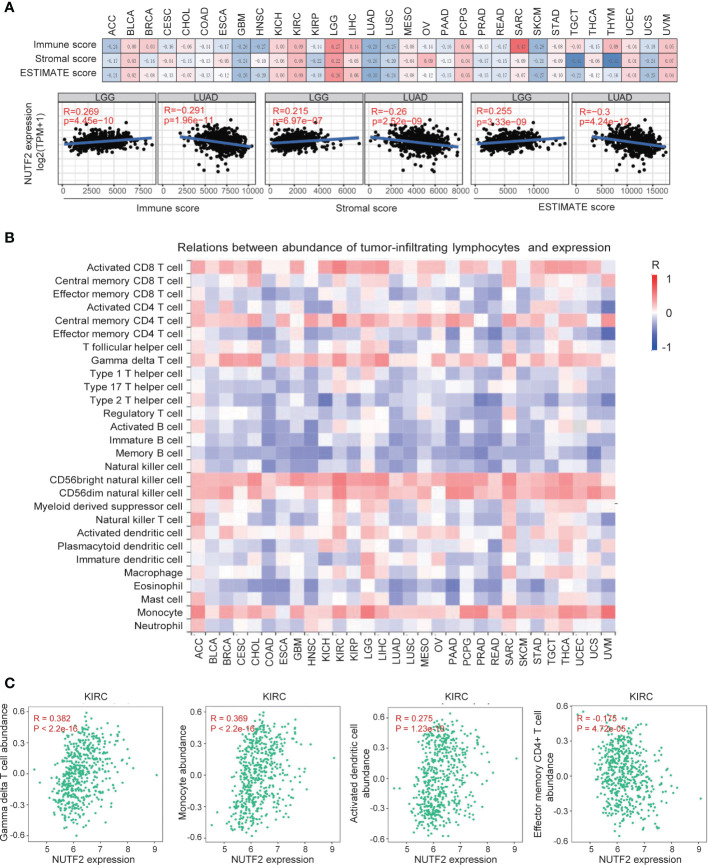
Correlation analysis between NUTF2 expression and the abundance of tumor-infiltrating lymphocytes. **(A)** The potential associations between infiltrating stromal and immune cells and NUTF2 expression level were explored by ESTIMATE algorithm. **(B)** Correlation analysis between abundance of tumor-infiltrating lymphocytes and NUTF2 expression in different cancer types. **(C)** Correlation between NUTF2 expression and Tgd, monocyte, activated dendritic cells, and Tem CD4^+^ cell infiltration in KIRC.

Malignant solid tumor tissue contains not only cancer cells, but also normal stromal, immune, epithelial, and vascular cells. It has been reported that tumor-associated stromal and immune cells play important roles in regulating tumor growth, metastasis, and drug resistance (28–32). In this study, we used the ESTIMATE algorithm (16) to calculate the potential association between the infiltrating stromal and immune cells and NUTF2 expression level. It was found that NUTF2 expression was significantly correlated with immune score, stromal score, and ESTIMATE score in several tumor types (**Figure 4A**). It is noteworthy that NUTF2 expression level is positively associated with immune score, stromal score, and ESTIMATE score in LGG (**Figure 4A**). Previous studies indicated that high immune/stromal/ESTIMATE scores were significantly correlated with poor prognosis and advanced tumor grade in LGG (33), suggesting the cancer promoting role of NUTF2 *via* facilitating stromal and immune cell infiltration in LGG. In LUAD, by contrast, low immune/stromal/ESTIMATE scores were correlated with poor survival and high-level tumor stage (34). Interestingly, NUTF2 expression was negatively associated with immune/stromal/ESTIMATE scores in LUAD (**Figure 4A**). These data suggest the potential role of NUTF2 in regulating the tumor microenvironment (TME).

In order to investigate the role of NUTF2 in regulating the interaction between tumor and immune cells, we used the TISIDB platform (17) to dissect the correlation between NUTF2 expression and infiltrating immune cells. In most cancer types, NUTF2 expression was positively associated with infiltrated activated CD8^+^ T cell, central memory CD4^+^ T cell, gamma delta T cell, CD56bright/CD56dim NK cell and monocyte (**Figure 4B**). In addition, we observed a statistically negative correlation of NUTF2 expression and estimated type 1/17/2 T helper cell, regulatory T cell, activated/immature/memory B cell, natural killer cell, and eosinophil cell infiltration (**Figure 4B**). Infiltrating immune cells perform distinct functions and different clinical impacts in cancers. In KIRC, the infiltration of adaptive immune subpopulation, including activated CD8^+^ T cells, Tem/Tcm CD8^+^ cells, and Tem CD4^+^ cells, showed anti-tumor activity and associated with good prognosis. By contrast, monocytes, regulatory T cells (Tregs), activated dendritic cells, and gamma delta T cells (Tgd) played a cancer-promoting role in KIRC (35). In the present study, we found that NUTF2 expression was positively associated with infiltrating Tgd, monocyte, and activated dendritic cells, while negatively correlated with Tem CD4^+^ cell infiltration in KIRC (**Figure 4C**). These results imply that NUTF2 promotes tumor progression by regulating Tgd, monocyte, activated dendritic cells, and Tem CD4^+^ cell infiltration in KIRC.

### Correlation Analysis Between NUTF2 Expression and Immune Cell Markers

**Figure 5:**
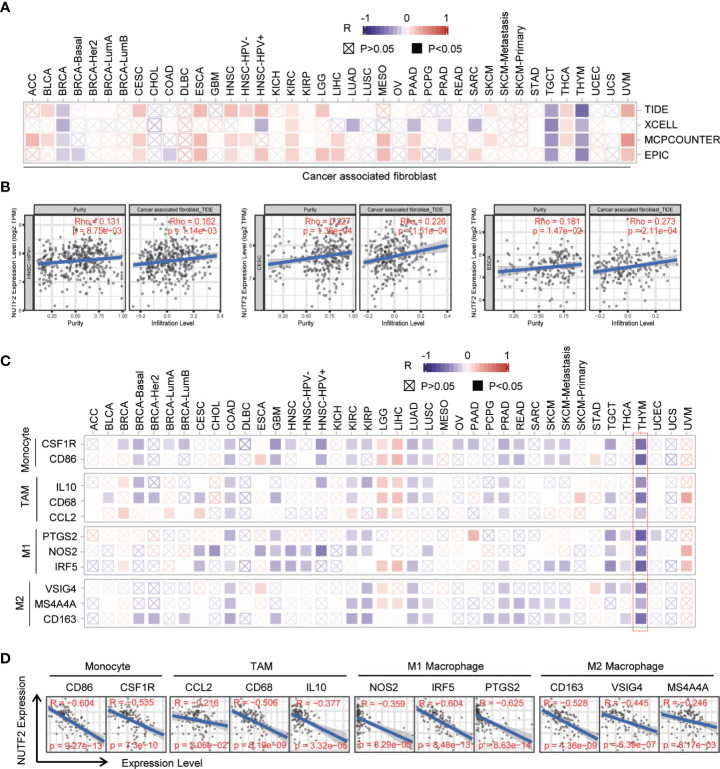
Correlation analysis between NUTF2 expression and immune cell markers. **(A)** The potential association between NUTF2 expression level and CAF infiltration was explored by different algorithms. **(B)** The correlations between NUTF2 expression and infiltrated CAF in BRCA, THCA, and THYM were analyzed by EPIC or TIDE algorithm. **(C)** Correlation analysis between NUTF2 expression and immune marker genes across all types of cancers in TCGA. **(D)** The correlations between NUTF2 expression and marker genes of monocyte, TAMs, M1 Macrophage, and M2 Macrophage in THYM.

Cancer-associated fibroblasts (CAFs), a kind of highly heterogeneous and hyper‐activated fibroblasts, have been demonstrated to promote tumor initiation, migration, inflammation, and drug resistance *via* the secretion of chemokines and cytokines, such as VEGFA and CXCL12 (36–38). In the present study, we used four different algorithms (TIDE, XCELL, MCPCOUNTER, and EPIC) to investigate the correlation between NUFT2 expression and infiltrating cancer-associated fibroblast. It was found that the number of infiltrating cancer-associated fibroblast was positively associated with the expression level of NUFT2 in CESC, ESCA, HNSC, KIRC, THCA, and UVM (appeared in at least 3 out of 4 algorithms) (*p* < 0.05) (**Figures 5A, B**). In addition, we also analyzed the relationship between NUTF2 expression and marker genes of immune cells, including monocyte, tumor-associated macrophage (TAM), M1 macrophage, and M2 macrophage. The results revealed that the expression of most markers of monocyte, TAM, M1 macrophage, and M2 macrophage were significantly associated with NUTF2 expression in COAD, KIRC, LGG, LUAD, PRAD, and THYM (*p* < 0.05) (**Figure 5C**). Specifically, it was found that CD86 and CSF1R of monocyte; CCL2, CD68, and IL10 of TAMs; NOS2, IRF5, and PTGS2 of M1 phenotype; and CD163, VSIG4, and MS4A4a of M2 phenotype were negatively correlated with NUTF2 in THYM (*p* < 0.05) (**Figure 5D**). Further investigation is needed to confirm the role of NUTF2 in regulating TME.

### Enrichment Analysis of NUTF2 Co-Expression Genes

**Figure 6:**
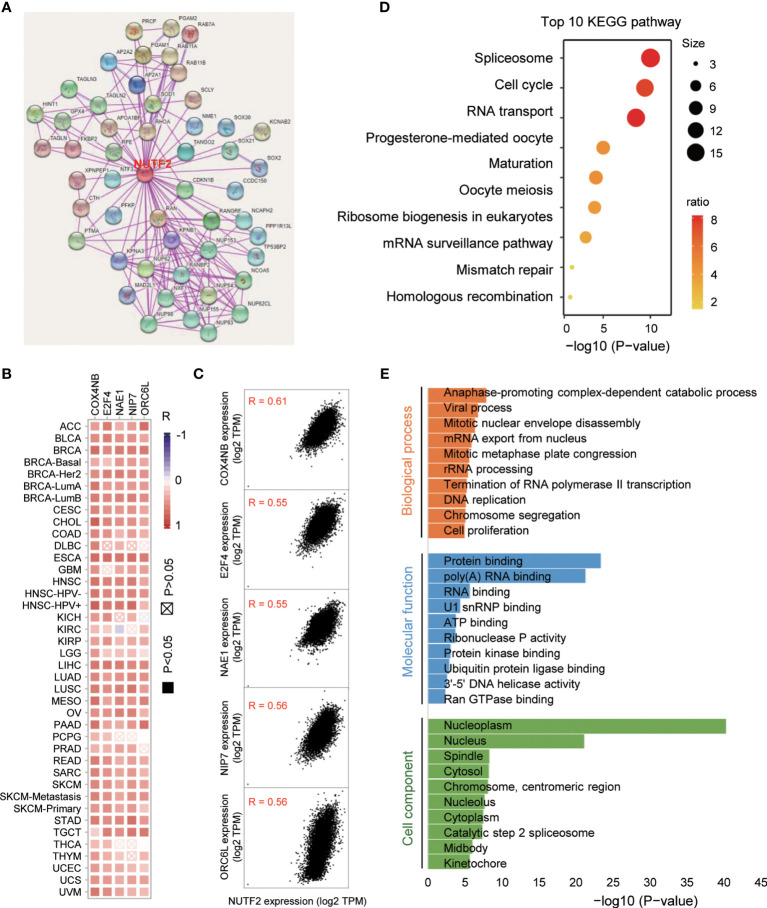
Enrichment analysis of NUTF2 co-expression genes. **(A)** Protein–protein interaction network was analyzed *via* the STRING online resource. **(B)** The heat map of correlation between NUTF2 and the top 5 genes in different cancer types. **(C)** Correlation of the top 5 genes and NUTF2 in all the cancer samples. **(D)** KEGG pathway analysis of the top 200 genes that associated with NUTF2 expression. **(E)** Gene ontology analysis of the top 200 genes.

In an attempt to investigate the potential molecular mechanism of NUTF2 in tumorigenesis, we performed the protein–protein interaction (PPI) network analysis *via* the STRING online tool. As shown in **Figure 6A**, a total of 50 NUTF2-binding proteins were obtained in the STRING dataset with experimental evidence (**Figure 6A**). Furthermore, we merged the expression data of all TCGA tumors and identified the top 200 genes that most associated with NUTF2 expression. The top 5 genes COX4NB (*R* = 0.61), E2F4 (*R* = 0.55), NAE1 (*R* = 0.55), NIP7 (*R* = 0.56), and ORC6L (*R* = 0.56) are shown in **Figure 6C**. In addition, the heat map revealed positive correlations between NUTF2 expression and the top 5 genes in the vast majority of cancer types (**Figure 6B**). Interestingly, recent studies showed that the higher expression of COX4NB, E2F4, and NAE1 was associated with poor prognosis in various cancers, suggesting its cancer-promoting role (39–43). Meanwhile, functional enrichment analysis of the top 200 genes identified a number of cancer-related pathways, such as spliceosome, cell cycle, and RNA transport (**Figure 6D**). We also focused on the gene ontology related to biological process, cell component, and molecular function. It was found that “anaphase-promoting complex-dependent catabolic process” and “protein binding” might be involved in the process of NUTF2 on cancer pathogenesis (**Figure 6E**). These results revealed the possible molecular mechanism of NUTF2 in tumorigenesis.

## Discussion

In this study, the expression of NUFT2 was investigated across the 33 types of cancers in the database of TCGA. Compared with corresponding adjacent normal tissues, the expression of NUTF2 was significantly upregulated in 19 cancer types of TCGA. Interestingly, we also observed a differential expression of NUTF2 between HNSC-HPV+ and HNSC-HPV-, evidently raising the potential association between HPV-related HNSC and NUTF2. Similar to tobacco and alcohol, human HPV infection was considered as a risk factor of HNSC (44). Compared with HPV-negative HNSC, the HPV-positive HNSC shows increased sensitivity to radiation and chemotherapy and better prognosis (45–48). It was reported that the tonsillar crypt epithelium is vulnerable to HPV infection and causes the integration of HPV DNA to host genome, resulting in the dysregulation of the oncoproteins E6 and E7 in host cells. The activation of E6 induces the degradation of p53, leading to the defects in DNA repair and causing genomic instability of the epithelium cells. Additionally, accumulated E7 protein interacts and inactivates the tumor suppressor RB, resulting in uncontrolled cell division and proliferation (49, 50). Compared with HPV-negative HNSC, the expression of NUTF2 is decreased in HPV-positive HNSC, raising the possibility that NUTF2 may be involved in the pathologic process of HPV-related HNSC *via* regulating the E6/E7 signaling pathways.

Breast invasive carcinoma (BRCA) was considered as a heterogeneous disease and can be divided into four classical subtypes based on the expression of ER, PR, and HER2, namely, HER2-enriched, basal-like, Lumina A, and Lumina B (51). Among these four subtypes, HER2-enriched and basal-like BRCA are more aggressive and have a worse prognosis than the other two subtypes (52). Interestingly, compared with Lumina A and Lumina B subtypes, the higher expression of NUTF2 was found in HER2-enriched and basal-like BRCA. Meanwhile, the Lumina A subtype displayed lower expression of cell proliferation-related genes and showed better prognosis as compared to the Lumina B BRCA (52). In our study, Lumina B tumors have higher NUTF2 expression than Lumina A. These results indicate that NUTF2 may play an important role in the progression and prognosis in breast cancer. To further investigate the role of NUTF2 in BRCA, we analyzed the associations between NUTF2 and the classic proliferation markers (MKI67 and PCNA) and EMT markers (VIM, TWIST1, SNAI1, SNAI2, FN1, and CDH2). It was found that the expression of NUTF2 was positively correlated with PCNA and MKI67 only in Lumina A tumors. Additionally, the expression of TWIST1, SNAI1, FN1, and CDH2 also showed significant association with NUTF2, suggesting that NUTF2 may play a major tumor-promoting role in the Lumina A BRCA subtype.

TME is a complicated and multilevel network of interactions between tumor cells and the surrounding components, including endothelial cells, fibroblasts, stromal cells, and immune cells. Benefiting from the development of next-generation sequencing technologies, the composition characteristics of infiltrated immune cells can be dissected in common cancers. Recently, Pornpimol and colleagues estimated 28 subtypes of infiltrated immune cells in 20 solid cancers *via* the GSEA strategy (35). Consistent with previous studies (53–55), the infiltration of activated CD8^+^ T cells and effector memory CD8^+^ T cells displayed anti-tumor effect and significantly associated with good prognosis in most types of cancers. By contrast, infiltrated MDSCs, Treg, and monotype showed a cancer-promoting role and correlated with poor survival. Additionally, the function of activated B cells and memory B cells varied in different cancer contexts. It was discovered that infiltration of these two types of immune cells exhibits a pro-tumor role in breast cancer, but shows anti-tumor effects and associated with satisfied prognosis in lung adenocarcinoma. The infiltrated immune cells function in a context-dependent manner, which means that a certain type of immune cell may display a beneficial prognostic effect in one cancer type but a harmful effect in another malignancy (56, 57). In the present study, we discovered that the expression of NUTF2 was negatively associated with the infiltrated MDSCs in HNSC, LUAD, LUSC, and SKCM. By taking the pro-tumor role of MDSCs in these four types of cancers into consideration, NUTF2-mediated MDSC infiltration may be a crucial cause for its oncogenic effect.

Cancer-associated fibroblasts (CAFs) are a subpopulation of hyper-activated fibroblasts within TME. It has been demonstrated that normal fibroblasts display inhibitory effects on the proliferation and motility ability of cancer cells (58). The exposure of normal fibroblasts to cancer-associated factors and TME stimulation, such as hypoxia stress, enhanced energy reprogram and activation of fibroblasts. Among numerous cancer-derived factors, IL6, TGFβ, and PDGF are the widely accepted fibroblast-activating factors that promote the activity of downstream signaling pathway, such as the SMAD and NF-κB signaling (59, 60). Compared with normal fibroblasts, cancer-associated fibroblasts express increased markers, such as FAP, PDGFRα, and αSMA, which have been used as biomarkers to isolate CAF population from the tumor tissue (36). It was reported that CAFs display a pro-tumorigenic effect and regulate tumor metastasis *via* secreting growth factors and remodeling the extracellular matrix (ECM), and are involved in tumor mechanics, drug resistance, angiogenesis, and inflammation (36). However, recent studies suggested that CAFs display phenotypic and functional heterogeneity. Heather and colleagues reported that two CAF subpopulations can be distinguished by the expression of CD146. The CD146^-^ CAFs inhibit the expression of estrogen receptor and response to estrogen, resulting in tamoxifen resistance, while the CD146^+^ CAFs provide durative estrogen-dependent proliferation and tamoxifen sensitivity of breast cancer cells (61). In our study, we found that the infiltration of CAFs was significantly associated with NUTF2 expression level in BRCA, TGCT, THYM, CESC, ESCA, HNSC, KIRC, THCA, and UVM (appeared in at least 3 out of 4 algorithms), suggesting that NUTF2 may participate in the transformation and activation of CAFs.

Gene dysregulation is a hallmark of cancer progression. Abnormal gene expression can be achieved in several ways, such as DNA mutation or copy number variation, promoter methylation, histone epigenetic modification, miRNA regulation, and m6A modification. In the present study, we revealed that the expression of NUTF2 was evaluated in 19 types of cancers. It was known that DNA copy number amplification and methylation were the two underlying causes for NUTF2 upregulation in cancers. However, the frequency of DNA amplification is relatively low in cancers, and other possible explanations for NUTF2 dysregulation need to be explored.

## Data Availability Statement

The original contributions presented in the study are included in the article/supplementary material. Further inquiries can be directed to the corresponding authors.

## Author Contributions

YL, YH, and JH contributed to conception and design of the study. YH organized the database. SR performed the statistical analysis. YL wrote the first draft of the manuscript. JH, XX, and HC wrote sections of the manuscript. All authors contributed to manuscript revision, read, and approved the submitted version.

## Funding

This research is supported by the National Natural Science Foundation of China (81903069 to YL, 81901641 to JH) and the Shenzhen Project of Science and Technology (JCYJ20210324110011031 to JH).

## Conflict of Interest

The authors declare that the research was conducted in the absence of any commercial or financial relationships that could be construed as a potential conflict of interest.

## Publisher’s Note

All claims expressed in this article are solely those of the authors and do not necessarily represent those of their affiliated organizations, or those of the publisher, the editors and the reviewers. Any product that may be evaluated in this article, or claim that may be made by its manufacturer, is not guaranteed or endorsed by the publisher.
